# Coping with clustering in sample size calculations

**DOI:** 10.1186/1745-6215-12-S1-A27

**Published:** 2011-12-13

**Authors:** Rebecca Playle, Mark Kelly, Robert Newcombe, Kerry Hood

**Affiliations:** 1 SEWTU, Cardiff University School of Medicine, Cardiff CF14 4YS, UK; 2 Dept of Primary Care and Public Health, Cardiff University School of Medicine, Cardiff CF14 4YS, UK

## Objectives

The objective of this study is to discuss some of the issues and give some examples of dealing with clustering in individually and cluster randomised trial sample size calculations.

## Background

Clustering often occurs in individually randomised trials and is sometimes ignored when calculating sample sizes. Examples include practitioner effects in individually randomised trials with more than one practitioner or therapist effects from group interventions where clustering may be in one arm only. Additional levels of clustering may also occur in cluster randomised trials and estimating their effects can be difficult.

## Methods

Sample size calculations for several recent grant applications submitted by the South East Wales Trials Unit (SEWTU) have attempted to account for clustering or reduce inter-practitioner variation in different ways.

## Results

Example 1: Clustering effects in an individually randomised trial of a group intervention for drug and alcohol detoxification based in prison were estimated for the intervention arm and the sample size of both arms inflated. Example 2: Clustering effects of anaesthetist were not estimated for a multi arm trial of 4 different airways devices but sample sizes were inflated to allow for small unknown effects. Sample size estimation used comparative and equivalence methods in three parallel multi arm trials. Example 3: Possible clustering in a trial of fissure sealant vs fluoride varnish at the school or family level was not accounted for in sample size calculations. All schools in the trial are Communities First schools and likely to be fairly homogeneous. Example 4: Unknown clustering effects of weight loss slimming groups were not accounted for in the intervention arm of a cluster randomised trials of a healthy lifestyle programme in obese pregnant women. Training has been implemented to reduce variability among group leaders.

## Conclusions

There appears to be no consensus regarding dealing with additional clustering effects and best efforts are made on a trial by trial basis for sample size estimation. Access to data for the estimation of intraclustering correlation coefficients and/or dissemination of the results will benefit all researchers designing trials with clustering issues.

